# The current status of health data on Korean children and adolescents

**DOI:** 10.4178/epih.e2017059

**Published:** 2017-12-26

**Authors:** Eunyoung Lee, Dahye Baik, Yoon Park, Moran Ki

**Affiliations:** Department of Cancer Control and Population Health, Graduate School of Cancer Science and Policy, National Cancer Center, Goyang, Korea

**Keywords:** Adolescent health, Child health, Health surveys, Korea

## Abstract

Childhood and adolescence are critical periods that affect adults’ health status. Therefore, the factors influencing the health of children and adolescents should be analyzed. In Korea, a wide range of youth-related health data has been obtained, both on the regional level and on the national level. This report summarizes the current status of studies related to the health of Korean children and adolescents. Data for which open access is offered include the Korea Youth Risk Behavior Web-based Study, the Panel Study on Korean Children, the Korean Youth Panel Survey, the Korean Children and Youth Panel Survey, and the Student Health Examination. In addition, the Health Examination of Korean Youth Outside of School, the Korean Children and Adolescents Obesity Cohort Study, the Korean Children’s Environmental Health Study, the Korea Youth Media Use and Harmful Environment Survey, the Comprehensive Survey of Korean Youth, and the Multicultural Adolescents Panel Study are summarized.

## INTRODUCTION

Childhood and adolescence are critical periods that affect adults’ health status and related habits, as well as being transitional periods of growth from childhood to adulthood. Therefore, the factors influencing the health of children and adolescents should be analyzed so that related indices can be developed for domestic and global comparisons. In our modern, dynamically changing society, proper tools are needed to diagnose changes in youth-specific conditions pertaining to physical and mental health; living circumstances, including home, school, and other public spaces; and any circumstances causing behavioral changes during early life.

Children and adolescents are continually growing, making it important to collect longitudinal health data from them, including biological samples [1]. The Trim and Fit Program in Singapore was an example of an active intervention program for children’s health in another country. This was a weight loss program administered through the Ministry of Education between 1992 and 2007 targeting students in elementary school through high school, and was identified by the World Health Organization as an effective method of improving children’s health [2]. The US, Japan, Denmark, and Norway have implemented large-scale birth cohort studies consisting of more than 100,000 participants that focus on environmental factors, although these cohort studies have different target follow-up ages [3].

In Korea, a wide range of youth-related data has been—and continues to be—obtained on the national level to promote the capacity of youth for development and healthy growth by providing them with various opportunities. To support youth properly, it is necessary to understand their health status and factors in their surrounding environment with direct, indirect, short-term, and long-term impacts on their lifespan. Based on a fundamental statistical analysis of the findings of youth surveys implemented through a dedicated surveillance system of adolescents in Korea, general (standard) youth indicators can be identified as a tool for establishing and developing youth policy. This report summarizes the current status of comprehensive surveys of Korean children and adolescents and the health-related data provided by these surveys.

There are 11 surveys covering the following broad topics related to health and wellness: physical health, health-related lifestyle factors, exposure to harmful environmental factors, social background, and individual development (Table 1). Results regarding children’s personal health status identified through health examinations and specific tests are included in the category of physical health, as well as individual and family medical histories. The category of health-related lifestyle factors includes the following subcategories: the use of substances, such as tobacco, alcohol, harmful chemicals, or drugs; sexuality, including information about sexual behaviors, partners, use of contraceptives, and experiences of pregnancy, abortion, and sexually transmitted infections; diet, including information about dietary habits and results from 3-day dietary assessments; physical activity, which refers to information about the frequency and extent of regular activity; weight control, which includes personal recognition and perceptions of the issue of weight and fitness; hygiene, which refers to the habits and cognitive skills needed to keep oneself clean (including dental hygiene); psychological status, which includes information on stress, emotions, and mental disorders; sleeping habits; health aids (e.g., the use of glasses); records of immunization and medication use (steroid, analgesic, antifebrile, antibiotics); and an allergy-related subcategory that investigates environmental health in youth. The category of exposure to harmful environmental factors includes the following circumstances that youth may encounter: media consumed through any online route, such as the Internet or mobile devices; exposure to nightlife establishments, such as pubs, karaoke establishments, and clubs; the indoor/outdoor environment, including exposure to environmental elements such as pollutants or toxic substances; safety and violence, including not only the awareness of being safe from direct harm, but also exposure to any kind of violence in any place; and radiation, which was specifically included in an environmental health study. The category of social background includes the following 3 separate dimensions of social life: domestic life, which refers to the home environment as well as relationships and any conflict with family members; school life, which includes any issues and circumstances that occur in school; and extracurricular life, which describes other ways that students spend their time, such as academic tutoring, physical activities, leisure, and part-time work. The category of individual development incorporates a wide range of developmental considerations influenced by the surrounding environment, such as physical growth, which pertains to the physical changes experienced during adolescence; intellectual development, which includes study habits and performance in school; social development, which includes self-perception and emotional stability; and career planning.

All of the surveys described in this study are ultimately related to the physical, mental, and behavioral health of youth (Table 2).

## MEASURES

### Korea Youth Risk Behavior Web-based Survey

The Korea Youth Risk Behavior Web-based Survey (KYRBS) has been conducted annually since 2005 to assess trends in health-risk behaviors among youth in Korea [4]. The target population of KYRBS is students in middle and high schools nationwide. Approximately 70,000 students are sampled from 400 middle schools and 400 high schools for the survey. It uses a multi-stage cluster sampling design, in which the sample was changed every 3 years from the beginning of the study through 2010, and every year since 2011.

The KYRBS has several strengths. It is an ongoing survey with nationwide samples of youth in Korea, and shows a very high response rate (over 95%) [5]. These high response rates have been possible because the survey is administered by the Ministry of Education, with students completing a web-based survey during class.

However, the KYRBS has some limitations. First, it is not representative of all youths, since the respondents are limited to students attending school. Second, the responses are self-reported, so participants may over-report or under-report their behaviors.

### Panel Study on Korean Children

The Panel Study on Korean Children (PSKC) is a long-term study tracking the growth process of Korean children. This is the first national panel study on newborn babies in Korea. It collects and provides, at a national level, cross-sectional and longitudinal data. It deals with data from the year 2008, when the panel children were born, to the year 2027, when they will be 19 years old. The PSKC includes data on children’s growth, development, childrearing environment, and experiences in child care centers, kindergartens, and schools.

The PSKC sampled 2,150 Korean babies born in 2008 using a stratified, multi-stage sampling technique, in which Korea was divided into the 6 districts of Seoul, Gyeonggi/Incheon, Chungcheong/ Gangwon, Gyeongbuk, Gyeongnam, and Jeolla. Then, medical institutions that deliver babies were identified, and families whose babies were delivered at those institutions were sampled.

The PSKC modifies its content annually, considering the children’s ever-changing growth, development, and environment. Since 2015, when the children entered primary school, PSKC has included content that is related to school life. By implementing performance tests to assess their development, the PSKC complements the weaknesses of regular questionnaires and explores the characteristics of children’s development more deeply [6].

### Korean Youth Panel Survey and Korean Children and Youth Panel Survey

The Korean Youth Panel Survey (KYPS) was a longitudinal survey carried out to identify and explain the behaviors and changing perceptions of adolescents. The Korean Children and Youth Panel Survey (KCYPS) was conducted after the KYPS. It included more panels and research content than the KYPS to study the diverse aspects of the growth and development of youth based on previous research experience [7].

The KYPS and KCYPS are among the major longitudinal studies that have been conducted in Korea and are considered to be comprehensive studies that provide an overview of the growth and development of Korean youth. These 2 surveys used a multiple-point prospective panel design, which involves multiple panels that are investigated at least twice at different times. This panel design has the characteristic of time continuity, which allows for causality and the developmental process of youth to be seen more clearly. During the survey, if a panel left the study, the study was continued by adjusting the panel with a weighted value.

The KYPS built a set of panel data by analyzing the changes in living patterns of youths after they entered middle/high school by following 2 panels (a panel in grade 4 of elementary school and a panel in grade 2 of middle school).

The KCYPS followed 3 panels (grades 1 and 4 of elementary school and grade 2 of middle school) for 7 years in order to include and observe the transitional periods of these students (elementary to middle school, middle school to high school, and high school to university). The KCYPS included many questions from various domains to ensure that the study was comprehensive. Questions for which the responses can change often, such as cell phone and internet use and adaptation to school life, were asked every year, whereas questions for which responses do not change very often, such as emotional development, self-esteem, and study habits, were asked every 2 or 3 years.

### Student Health Examination

The legal basis of the Student Health Examination is the Articles 7, 7-2, and 7-3 of the School Health Act. The School Physical Examination Regulation was established in 1951. The School Health Act was transformed from a physical examination system to a health screening system in 2005 [8].

The current Student Health Examination, which is conducted as a sample survey, started in 2009. Survey subjects are sampled in increments of whole school years from elementary school to high school for the physical development and health survey, and in 4 groups defined according to the school year (grades 1 and 4 in elementary school, grade 1 in middle school, and grade 1 in high schools) for the health check-up.

Stratified cluster sampling is applied by 17 cities and provinces throughout Korea for the allocation of samples. Health check-ups are carried out at selected institutions, including examinations of the musculoskeletal system, eyes, ears, nose, skin, and oral cavity, as well as tests for various pathologies. A questionnaire-based health survey investigates health behaviors including dietary habits, sleep, and physical activity. Distinct questionnaires are used for elementary school students and for middle and high school students.

The Student Health Examination has the strength of being representative of students across the country and of being regularly conducted, enabling continuous trends to be observed. Limitations include the fact that health check-ups are conducted only for students in 4 grades, including a limited range of conditions, and the cross-sectional nature of the data.

### Health Examination of Korean Youth Outside of School

The Health Examination of Korean Youth Outside of School is a national support service that has been conducted for self-supporting youth since 2016. The legal basis of this health examination is the Act on Supporting Out-of-School Youth enacted in 2014. The organization that conducts these examinations, K-dream, is supported by the Ministry of Gender Equality and Family and the Korea Youth Counselling and Welfare Institute, and contributes to the monitoring of the health status of out-of-school youths by collecting health data [9].

The targeted subjects are defined as any youth aged 9 to 18 who has been absent over 3 months after the entrance or postponement of schooling; who has been expelled, withdrawn, or has dropped out for oneself; and who has not been promoted to an advanced school. Once the National Health Insurance Service (NHIS) confirms that an applicant is qualified for this health examination, the subject can visit an NHIS online system known as HealthiN (https://hi.nhis.or.kr) to search available designated examination centers.

In the health examination, a questionnaire is administered before the medical check-up items are assessed. If necessary, examinees can receive counseling from a medical specialist for concerns including mental health.

### Korean Children and Adolescents Obesity Cohort Study

This cohort started with first graders in 4 elementary schools in the city of Gwacheon in 2005. Continuous follow-up is in progress after expansion of the cohort to include 7 more schools in the Junggu area in Seoul and the southwestern area of Gyeonggi province. A cohort with morbid obesity was additionally established from 2012 to 2014.

In this cohort, sexual maturity ratings are obtained and dietary assessments for 3 days by 24-hour recall are conducted. The questionnaire includes items on topics such as dietary habits, health status, exercise, familial environment, and weight management.

The Korean Children and Adolescents Obesity Cohort Study collects longitudinal data and provides evidence to identify risk factors for pediatric obesity. However, it is geographically limited to a few areas and is not wholly representative of the pediatric population.

### Korean Children’s Environmental Health Study

This study is based on Article 15 of the Environmental Health Act. It consists of a main cohort, which is used to calculate the incidence of diseases, and a core cohort, which is focused on identifying the causes of disease. It aims to recruit 100,000 pregnant women, including 70,000 pregnant women over the 5-year period starting in 2015. In the core cohort, a 20-year follow-up is conducted.

In the main cohort, questionnaires for pregnant women, collection of biological specimens, clinical tests, and follow-up examinations of infants are carried out. In the core cohort, questionnaires for pregnant women, assessment of the surrounding environment, analysis of environmental pollutants through blood tests and urinalysis, health check-ups of pregnant women, genetic tests to identify association between genes and environmentally-linked diseases, a survey of delivery outcomes, and follow-up examinations of infants are conducted.

The Korean Children’s Environmental Health Study is a prospective cohort study that follows up children and investigates their exposure to hazardous environmental factors and the effects of those factors on their health from infancy to adolescence, in order to identify causal relationships between the exposure and disease and to establish preventive solutions.

### Korea Youth Media Use and Harmful Environment Survey

The Korea Youth Media Use and Harmful Environment Survey was specifically designed to monitor youth’s environment, with a focus on exposure to harmful factors that may threaten their safety or affect their behavior. The samples of this dataset inclusively comprise school students and juveniles in crisis, such as runway adolescents who have registered at designated shelters or juvenile delinquents under probation or in prison.

### Comprehensive Survey of Korean Youth

In contrast to studies with specific topics, the Comprehensive Survey of Korean Youth focuses on monitoring trends in various domains that may affect youth life with a relatively large scope. This survey also collects data from the caregivers or parents who live together with the youth respondent. It provides information that is useful for understanding youth because the role of parents and the domestic atmosphere may strongly affect the development of youth as they grow up. Since the sampling unit is a household with a residential address, the samples are composed of school students and youth outside of school.

### Multicultural Adolescents Panel Study

As the number of multicultural families grows in Korea, there is a need to develop and increase the capacity to accept multiculturalism and to integrate these families into Korean society. Based on this need, the Multicultural Adolescents Panel Study (MAPS) was established to investigate the development of adolescents with a multicultural background [10].

The MAPS started in 2010 as preliminary research and its first stage of research was completed in 2012. The objective was to collect longitudinal data on multicultural adolescents. Starting in 2013, the MAPS has conducted its second stage, extending through 2017 [10]. The objective of this stage was to follow multicultural adolescents and to identify changes in their development and the causes of them. Although the objectives of the 2 stages were different, this study can be considered to be a consecutive study since the initial panels were the same.

Since 2011, the MAPS has conducted surveys and focus group interviews with its panels. For the interviews, pairs of multicultural adolescents and their mothers were selected from 4-5 households among the survey participants from different regions.

## DATA ACCESSIBILITY

Open-access data are listed in Table 3. Raw data can be obtained from the website of the administrative agency that has ownership of the data. Not all data have open accessibility. However, statistical summaries and analyses of all data are provided in periodic reports released to the public.

## Figures and Tables

**Table 1.** Health-related surveys of Korean youth

Surveys	Physical health					Health-related lifestyle factors												Exposure to harmful environmental factors					Social background			Individual development			
	Medical check-up	Medical history	Sexual maturity rating	Genetic testing	Follow-up of infants	Use of substances	Sexuality	Diet	Physical activity	Weight control	Hygiene	Psychological status	Sleeping habits	Health aids	Records of immunization	Medicines	Allergy-related	Media/Internet/Mobile	Nightlife establishment	Indoor/Outdoor environment	Safety/Violence	Radiation	Domestic life	School life	Extracurricular life	Physical growth	Intelligence	Social/Cultural/Emotional	Career planning
Korea Youth Risk Behavior Web-based Survey																													
Panel Study on Korean Children																													
Korean Youth Panel Survey																													
Korean Children and Youth Panel Survey																													
Student Health Examination																													
Health Examination of Korean Youth Outside of School																													
Korean Children and Adolescents Obesity Cohort Study																													
Korean Children’s Environmental Health Study																													
Korea Youth Media Use and Harmful Environment Survey																													
Comprehensive Survey of Korean Youth																													
Multicultural Adolescents Panel Study																													

**Table 2.** Overview of health-related surveys/databases of youth

	Korea Youth Risk Behavior Web-based Survey	Panel Study on Korean Children	Korean Youth Panel Survey	Korean Children and Youth Panel Survey	Student Health Examination	Health Examination of Korean Youth Outside of School
Purpose	To investigate health-risk behaviors of Korean youth and to monitor their behaviors to help them build healthy habits	To provide data about the development of Korean children and to contribute to childcare and education policies for their wellbeing	To identify changes in behaviors and perceptions in adolescence, and the causes of these changes by observing the same panels during their growth	To investigate comprehensive aspects of physical and psychological changes in children and youth, and the causes of these changes by observing the same panels during their growth	To analyze student health, including physical development and disease status, and to develop a credible index of student health	To support youths outside of school by monitoring their health status for the prevention and early detection of diseases during their development
Survey period	Since 2005	From 2008 to 2027	From 2004 to 2008 for grade 4 of elementary school	From 2010 to 2016	Since 2006	Since 2016
	Annually	Annually	From 2003 to 2008 for grade 2 of middle school	Annually	Annually	Once every 3 yr (if the status of a subject remains an out-of-school juvenile)
			Annually			
Geographic areas	Nationwide, 17 cities and provinces	Nationwide, 6 zones across the country	Nationwide, 12 cities and provinces	Nationwide, 16 cities and provinces	17 cities and provinces across the country	Nationally, 514 designated detection centers
Population sampled	Students in middle and high school	Newborn babies in 2008	Fourth-graders in elementary school and their parents	First-graders and fourth-graders in elementary school and their parents	Students in all grades in elementary, middle, and high schools	Out-of-school juveniles who register and sign up for the health examination and screening program
			Children in the second grade of middle school and their parents	Children in the first grade of middle school and their parents		
Sample size range	As of 2016,798 schools 65,528 students	2,150 newborn babies across the country	As of 2004,2,844 people (elementary school grade4 panel)	As of 2010, elementary school grade 1 panel: 2,342 people; elementary school grade 4 panel:	765 schools across the country (2016) 82,883 students (survey and physical development)	As of 2016,6,986 non-institutionalized juveniles outside the school system
			As of 2003,3,449 people (middle school grade 2 panel)	2,378 people; middle school grade 1 panel: 2,351 people	27,671 students (health check-up, grades 1 and 4 of elementary school, grade 1 of middle and high schools)	
Representative	Weighted to be representative of the population of Korean youth	Stratified multi-stage sampling	Weighted to be representative of Korean youth of each panel grade, with a lapse in time from the base grade in the first year	Weighted to be representative of Korean children and youth of each panel grade, with a lapse in time from the base grade in the first year	Stratified cluster sampling across 17 cities and provinces	Not representative
Methods	Survey managed by trained teachers	Questionnaire	Repeatedly investigating the survey for the same panels	Repeatedly investigating the survey for the same panels	Physical development	Self-administered questionnaire before health check-up
	Anonymously completion of self-administered questionnaires	Computer-assisted personal interview through a home visit	For youth: group interview in school, individual interview by following up with each person	For youth: group interview in school, individual interview by following up with each person	Survey	Blood, hepatitis testing, tuberculosis testing, and dental condition
		Observation, interview, and testing (in-depth study)	For parents: telephone interview	For parents: telephone interview	Health check-up	
Survey contents (topics)	Analysis of behaviors regarding health-related status, attitudes, and perceptions in Korean youth	Characteristics of children, parents, and family	Changes of lifestyle and attitudes toward occupational choice, career planning, deviance and so on, which are developing in youth (longitudinal survey)	Individual developments, including physical and emotional changes and development, environment at home, among peers, and in the community observed in childhood and adolescence (longitudinal survey)	Current status regarding physical development, including body mass index and degree of obesity	Health check-ups suitable for life transitions at a young age
		Characteristics of childcare and education services			Health behaviors such as diet, physical activity, and safety awareness	Current status regarding physical development and basic medical condition
		Characteristics of local community and child care policies			Health status including eyesight, oral, and skin disease	Counseling with a professional if needed

	**Korean Children and Adolescents Obesity Cohort Study**	**Korean Children’s Environmental Health Study**	**Korea Youth Media Use and Harmful Environment Survey**	**Comprehensive Survey of Korean Youth**	**Multicultural Adolescents Panel Study**	

Purpose	To follow up a pediatric obesity cohort until adolescence and to analyze risk factors for childhood obesity and accompanying metabolic diseases	To provide a basis for developing a list of hazardous environmental factors for each stage of growth and a scientific foundation for the relationship between environment-related diseases and harmful environmental factors	To investigate the harmful factors affecting Korean youth and to identify the status of harmful exposures that may interrupt their healthy growth	To investigate diverse factors related to health, everyday life, and social and cultural experiences of Korean youth	To follow the development of multicultural adolescents, to identify factors associated with changes in their behavior and thinking, and to contribute to reducing the gap between multicultural and non-multicultural adolescents	
Survey period	Since 2005 and 2012 (morbid obesity cohort)	Since 2015 (for 22 yr)	Since 1999	Since 2011	From 2010 to 2017	
	Annual follow-up		Biennially (even-numbered years)	Once every 3 yr	Annually	
Geographic areas	3 regions (Gwacheon city in Gyeonggi province, Jung-gu in the city of Seoul, and the southwestern area of Gyeonggi province)	Through invitations for 5 yr starting in 2015, considering the birth rate in metropolitan cities and provinces across the country	Nationwide, all 18 cities and provinces	Nationwide, all 18 cities and provinces	Nationwide, 16 cities and provinces	
Population sampled	Follow-up of first-graders in elementary school since 2005	Pregnant women invited from obstetrics/gynecology clinics and public health centers	Students in school: elementary grades 4-6 and all grades of middle and high school	Samples of caregiver(s) and children aged 9-24 who are living with their caregiver(s) at registered residential address	Multicultural fourth grade adolescents in elementary school and their parents (especially mothers)	
			Non-institutionalized juveniles in crisis			
Sample size range	4,580 students, including the morbid obesity cohort (2014)	5,000 pregnant women for the core cohort (for 3 yr)	As of 2016,15,646 students (elementary to high school) 1,876 juveniles in crisis (ages, 12-19 yr)	As of 2014, based on 2,000 residential units: 3,000 children aged 9-24:2,000 caregivers living with the sample children	As of 2011,1,625 households by the sampling unit of schools (first) and all multicultural fourth-grade students and their mothers (second) in the selected schools	
		65,000 pregnant women for the main cohort (for 4 yr)				
Representative	Not representative	Nationwide network of recruiting offices	Weighted to be representative of the population of Korean juveniles	Weighted to be representative of the population of Korean youth living with caregiver(s) in the same residential unit	Weighted to be representative of the population of multicultural adolescents and parents who live in Korea	
Methods	Questionnaire	Questionnaire	Survey managed by a trained investigator	Survey managed by a trained interviewer who visits the sample family	Survey investigating the same panel repeatedly, managed by a trained investigator	
	Dietary assessment (3 d)	Environmental measurement	For students: self-administered questionnaire	Separate survey for caregiver and youth (ages 9-12,13-24 yr)	Panel questionnaire survey	
	Health check-up	Clinical tests	For juveniles in crisis: interview	Self-administered questionnaire, if needed	Focus group interview	
	Collection of biological samples	Genetic tests				
		Follow-up of infants				
Survey contents (topics)	Comparison of health status, including body composition and sexual maturity, between obese and normal children	Analysis of exposure of pregnant women to hazardous environmental factors and the effect thereof on infants' health	Youth exposure to harmful media and environment that can possibly affect lifestyles and social behaviors (time-serial comparison)	Youth’s social/cultural background, health-related life behavior, and emotional status (time-serial comparison)	Multicultural variables including language and background, individual and environmental characteristics of multicultural adolescents (longitudinal survey)	
	Follow-up of children with morbid obesity					

**Table 3.** Data accessibility

Surveys	Administrative agency (ownership of data)	Periodic report	Open accessibility	Web address/procedure
Korea Youth Risk Behavior Web-based Survey (KYRBS)	Korea Centers for Disease Control and Prevention (KCDC)	Yes	Yes	https://yhs.cdc.go.kr
				① Go to KYRBS website → utilizing result → application for the use of primitive data
				② Enter email and password → write down the requisition form
				③ Check the status of application → download (maximum 3 times)
Panel Study on Korean Children	Korea Institute of Child Care and Education (KICCE)	Yes	Yes	http://panel.kicce.re.kr
				① Make research proposal
				② Submit proposal to http://kicce.re.kr/panel
				③ Obtain KICCE’s approval
				④ Download data
Korean Youth Panel Survey (KYPS)	National Youth Policy Institute (NYPI)	Yes	Yes	http://archive.nypi.re.kr
				① Go to NYPI website → Data Archive
				② NYPI Panel Survey → KYPS → Questionnaire/Code book/Data
Korean Children and Youth Panel Survey (KCYPS)	NYPI	Yes	Yes	http://archive.nypi.re.kr
				① Go to NYPI website → Data Archive
				② NYPI Panel Survey → KCYPS → Questionnaire/Code book/Data
Student Health Examination	Ministry of Education	Yes	Yes	http://www.open.go.kr
				① Go to “Open a request”
				② Go to “Apply for a request”
				③ Log in by creating an account
				④ Fill out the request form, designate the receiving institution as the Ministry of Education, and submit it → reply by e-mail
Health Examination of Korean Youth Outside of School	Ministry of Gender Equality and Family	Yes	No	—
Korean Children and Adolescents Obesity Cohort Study	KCDC	Yes	No	—
Korean Children's Environmental Health Study	Ministry of Environment	Yes	No	—
Korea Youth Media Use and Harmful Environment Survey	Ministry of Gender Equality and Family	Yes	No	—
Comprehensive Survey of Korean Youth	Ministry of Gender Equality and Family	Yes	No	—
Multicultural Adolescents Panel Study	NYPI	Yes	No	—
