# Supplementary material for: The current status of health data on Korean children and adolescents
Source: Epidemiol Health. 2017 Dec 26;39:e2017059. doi: 10.4178/epih.e2017059 (PMC5847967; doi:10.4178/epih.e2017059)
Supplement: Supplementary file 1 [file epih-39-e2017059-supplementary.pdf]

Volume: 39, Article ID: e2017059

https://doi.org/10.4178/epih.e2017059 [텍스트 입력]

한국청소년 건강관련 데이터 현황

이은영[1], 백다혜[1], 박윤[1], 기모란[1,*]

[1]암관리학과, 국제암대학원대학교, 국립암센터

초록

청소년시기는 성인의 건강상태에 영향을 미치는 중요한 시기이다. 따라서 청소년의 건강에 영향을 미치는 여러 가지 요소들에 대한 분석이 필요하다. 한국에서 청소년건강과 관련한 데이터는 지역 단위에서뿐 아니라 국가 단위에서도 수집되고 있다. 해당 데이터가 공개되고 있는 것으로는 청소년건강행태온라인조사, 한국아동패널, 한국청소년패널조사, 한국아동・청소년패널조사, 학교건강검사가 있다. 이외에도 학교 밖 청소년 건강검진조사, 소아비만 및 대사질환 코호트, 어린이 환경 보건 출생코호트, 청소년 매체이용 및 유해환경 실태조사, 청소년 종합실태조사, 다문화청소년패널조사를 요약하였다.

서론

아동기와 청소년기는 아동이 성인으로 이행하는 시기일 뿐 아니라 건강 상태와 습관을 형성해가는 데 영향을 미치는 중요한 시기이다. 그러므로 아동과 청소년의 건강에 영향을 주는 다양한 요인들을 분석하는 것이 필요하다. 급격하게 변화하는 사회에서, 아동・청소년의 신체 및 정신건강, 생활환경, 행동에 영향을 끼칠 수 있는 여러 배경 등 다양한 측면을 포함한 그들의 특정한 상태의 변화를 진단할 수 있는 적절한 도구가 요구된다.

아동과 청소년의 성장은 계속되고 있으며, 단면연구뿐 아니라 종단연구를 통해서도 그들의 연속적인 건강 관련 데이터를 수집하는 것이 필요하다. 아동의 건강 관련 정책에 적극적인 해외의 사례를 보면, 싱가포르의 Trim & Fit 프로그램이 있다. 이 프로그램은 교육부 주도하의 체중 감소 프로그램으로, 1992년부터 2007년까지 초등학생에서 고등학생을 대상으로 시행되었다. 또한 이 프로그램은 WHO로부터 아동의 건강 향상에 효과적인 방법 중 하나로 선정되었다. 미국, 일본, 덴마크, 노르웨이는 십만 명 이상의 규모가 큰 출생코호트를 진행하고 있다. 각각의 follow-up 대상 나이는 다르지만, 주로 환경적인 요인에 중점을 두어 연구한다.

한국에서는 청소년의 역량 발달을 촉진시키고 성장에 다양한 기회를 제공하기 위해 국가

https://doi.org/10.4178/epih.e2017059

수준의 조사를 시행해 청소년 건강 관련 데이터를 확보하고 있다. 청소년에 대한 적절한 지원을 위해서는 그들의 건강상태와 주변의 직간접적, 장단기적인 영향을 주는 환경에 대한 이해가 요구된다. 청소년 조사들을 기반으로 한 표준 청소년 지표들은 청소년 정책을 수립하는 데 기초적인 통계자료로 사용될 수 있다. 이 보고서는 우리나라의 아동과 청소년에 대한 포괄적인 조사와 건강 관련 데이터의 현 상황을 밝히고자 한다.

(Table 1)

건강과 관련된 다양한 주제들(신체 건강, 건강 관련 생활방식, 유해 환경에의 노출, 사회적 배경, 개인 발달)을 연구한 11개의 조사가 있다 [Table 1]. 개인과 가족의 병력뿐 아니라 건강 검진과 특정 검사에 의한 개인의 건강 상태가 ‘신체 건강’ 항목에 해당된다. ‘건강 관련 생활방식’ 항목에는 다양한 하위 항목이 포함된다. 유해 물질의 사용(흡연, 음주, 약물), 성행태(성적 행동, 피임도구의 사용, 임신 경험, 낙태, 성병), 식생활(식습관, 3일 식이조사 결과), 신체 활동(정기적인 활동, 개인적 인식에 의한, 체중과 체력에 대한 스트레스로 인한 체중 조절), 구강 검진을 포함한 개인의 위생 관리와 인식, 스트레스, 정서, 정신 장애와 관련된 정신건강, 수면습관, 건강 보조도구(안경착용), 예방접종(횟수), 약제사용(스테로이드 연고 사용기간, 진통・해열제 복용, 항생제 치료) 그리고 알레르기와 관련된 하위 항목들이 조사되었다. ‘유해 환경에의 노출’ 항목에는 청소년이 경험할 수 있는 다양한 가능성을 조사했다. 인터넷, 모바일 등 모든 온라인 활동을 포함한 미디어의 사용, 유해 업소에의 노출(술집, 노래방, 클럽), 오염 물질 및 독성 물질에의 노출과 관련된 실내외 환경, 안전에 대한 인식과 폭력에의 노출과 관련된 안전과 폭력 그리고 환경적인 건강 조사에 특별히 포함된 방사능이 하위 항목으로 구분된다. ‘사회적 배경’ 항목은 3가지 측면으로 나누어져 있다. 가정 환경 및 가족간의 관계와 갈등과 관련된 가정, 학교 그리고 학원, 신체 활동, 여가, 아르바이트 등의 교외 활동이 이에 해당 된다. ‘개인 발달’ 항목에는 청소년이 자신을 둘러싼 주변환경의 영향을 받으면서 성장해가는 양상을 설명했다. 청소년기에 겪는 신체 변화 관련된 신체 발달, 학습 습관이나 성적과 관련된 지적 발달, 자아 인식 및 정서 안정과 관련된 사회・정서적 발달, 진로계획이 이에 해당 된다.

모든 조사는 이러한 주제를 기반으로, 신체 및 정신 건강 그리고 건강 관련 행동을 궁극적으로 조사하였다 [Table 2].

(Table 2)

청소년건강행태온라인조사

청소년건강행태온라인조사는 우리나라 청소년의 건강행태 현황과 추이를 파악하기 위해

Volume: 39, Article ID: e2017059

https://doi.org/10.4178/epih.e2017059 [텍스트 입력]

2005년부터 매년 시행되고 있다. 전국의 중학교 및 고등학교 재학생을 목표 모집단으로 하여 중・고등학교 각각 400개 학교를 선정한 후 임의로 한 학급씩 표본학급을 뽑아 약 7만명의 학생을 조사했다. 표본추출은 층화, 군집, 다단계 표본추출의 과정을 거친 복합 표본 설계를 사용했다. 2010년 조사까지는 표본을 3년 주기로 교체했지만, 2011년부터 매년 표본을 새로 추출하고 있다.

청소년건강행태온라인조사는 청소년을 대상으로 계속해서 진행되고 있는 전국 단위 조사이고, 95% 이상의 높은 응답률을 보인다는 점에서 강점을 가지고 있다. 이러한 높은 응답률은 교육부 주도하에 온라인 기입식 조사를 학교 수업 중에 일괄적으로 시행하기 때문에 가능하다.

청소년건강행태온라인조사의 한계점은 첫째로, 학교에 다니고 있는 학생을 대상으로 하기 때문에 모든 청소년을 대표하지는 못한다는 점이다. 둘째로, 자기보고식 조사이기에 과소 혹은 과장하여 응답할 수 있다는 점이다.

한국아동패널

한국아동패널은 종단연구를 통해 전반적인 발달궤적을 추적하여 한국 아동들의 내・외적 변인들을 광범위하고 체계적으로 살펴본 연구이다. 이 연구는 2008년 4월부터 7월 사이에 태어난 신생아 가구를 대상으로 일반조사와 심층조사를 통해 이루어진다. 전화 조사와 자필조사, 면접 및 관찰 등의 다양한 연구조사 방법을 활용했다. 또한 횡단적 자료를 구축하기 위하여 부가조사를 통해 2010년 출생한 아동들의 특성과 추이를 파악했다. 2015년 8차조사에서는 알레르기 질환에 대한 정밀 자료 수집을 위해 병원조사를 실시했다. 또 아동의 발달을 고려하여 연령의 증가에 따라 아동의 학교생활과 관련된 변인과 교사의 응답 등이 추가되었다. 이런 포괄적인 데이터를 통해 아동에 대한 깊은 이해와 정책적인 제안이 가능하므로 이를 활용하여 아동의 건강한 성장을 도모할 수 있다.

한국청소년패널조사 & 한국아동청소년패널조사

한국청소년패널조사는 청소년 행위의 실태 및 변화양상에 대해 파악하고 그 원인을 설명하기 위한 종단 조사이다. 한국아동・청소년패널조사는 청소년패널조사의 후속연구로 이전 연구 경험을 바탕으로 조사대상과 연구내용을 보다 확대하여 아동・청소년 성장과 발달의 다양한 양상을 종합적으로 파악하는 것을 목적으로 조사를 진행했다.

한국청소년패널조사와 한국아동・청소년패널조사는 청소년 성장과 발달의 전체상을 연구한 국내의 대표적인 종단조사이다. 두 조사 모두 동일한 표본을 적어도 두 번 이상 서로 다른 시점에서 조사하는 multiple point prospective 패널 설계를 사용했다. 이런 패널 설계

는 시간의 연속선상에서 아동・청소년의 성장과 발달 과정을 관찰해 시간의 경과에 따른 변인간 인과관계 설명에 용이하다. 조사 기간 중 표본이 상실될 경우, 표본을 대체하지 않고 가중치를 부여하여 표본의 대표성을 유지하였다.

한국청소년패널은 초등학교 4학년과 중학교 2학년 패널을 추적하여 청소년이 중・고등학교 진학 시에 겪는 변화된 생활실태를 조사했다.

한국아동・청소년패널조사는 세 가지 패널(초등학교 1학년 및 4학년, 중학교 2학년)을 7년 동안 추적했다. 이를 통해 학생들이 초등학교에서 중학교, 중학교에서 고등학교, 고등학교에서 대학교로의 이행하는 과정을 포함하고 관찰하고자 했다. 한국아동・청소년패널조사는 다양한 영역을 포괄적으로 연구해 조사 문항수가 타 청소년 관련 연구에 비해 많다. 휴대폰 사용, 미디어 이용, 학교생활적응 등 가변성이 높은 항목은 매년 조사한 반면, 정서문제, 자아존중감, 학습습관 등 가변성이 낮고 상대적으로 안정적인 항목은 2년 또는 3년 주기로 조사했다.

학교건강검사

학교건강검사는「학교보건법」제 7조 및「학교건강검사규칙」에 법적 근거를 둔다. 학교신체검사규정은 1951년에 제정되었고 2005년 「학교보건법」 개정으로 신체검사가 건강검사로 전환되었다.

표본조사로 진행되는 현재의 학교건강검사는 2009년에 시작되었다. 조사대상자는 초등학교부터 고등학교까지 전 학년에 걸쳐 시행되는 신체발달현황 및 건강조사와 4개학년(초등학교 1학년과 4학년, 중학교 1학년과 고등학교 1학년)을 대상으로 시행되는 건강검진으로 크게 구분할 수 있다.

17개 시도로 구분하여 층화집락추출법에 따라 표본학교가 선정되고 학년별 1개 표본학급을 추출하여 표본학생을 추출한다. 건강검진은 지정된 건강검진기관에서 시행되고 근골격 및 척추, 눈과 귀, 콧병, 목병, 피부병, 구강, 병리검사 등이 포함된다. 건강조사는 구조화된 건강조사표를 통해 식습관, 수면, 운동 등 건강행태와 생활습관 등을 조사한다. 건강조사표는 초등학교용과 중고등학교용으로 구별된다.

학교건강검사는 전국의 학생을 대표하고 조사가 정기적으로 이루어져 지속적인 추세가 관측될 수 있다는 강점이 있다. 한계점은 건강검진이 일부 학년에서 특정 조건에 따라 시행되고 단면적인 데이터라는 점이다.

학교 밖 청소년 건강검진조사

Volume: 39, Article ID: e2017059

https://doi.org/10.4178/epih.e2017059 [텍스트 입력]

학교 밖 청소년 건강검진조사는 2016년에 신규 도입된 학교 밖 청소년지원사업의 한 종류이다. 2014년 학교 밖 청소년 지원에 관한 법률이 제정됨에 따라 시행되었다. 여성가족부와 한국청소년상담복지개발원에서 지원하는 꿈드림(K-drearm)에서 사업을 수행하고 있으며, 데이터 구축으로 학교 밖 청소년들의 건강관리 체계를 마련하고자 한다.

해당 검진사업은 정규학업을 중단하고 자립해야 하는 청소년들을 지원하는 학교 밖 청소년 지원사업 중 하나이다. 서비스 대상은 9세 이상 18세 이하의 청소년으로, 정규교육학교에 입학 후 3개월 이상 결성하거나 취학의무를 유예한 청소년, 제적이나 퇴학 처분을 받거나 자퇴한 청소년, 또는 상급학교에 진학하지 않은 청소년에 해당한다. 국민건강보험공단에서 적합한 건강 검진자에 대한 확인을 하고, 해당 검진자는 건강iN 시스템(https://hi.nhis.or.kr)에 접속하여 지정된 검진센터를 찾아 방문하면 된다.

건강검사에 앞서 대상 청소년들의 건강상태를 미리 알아보고 진찰을 받을 때 참고하기 위한 문진표를 작성하며, 각 대상자들의 정신건강 등의 특이점을 고려한 상담을 진행한다.

소아비만 및 대사질환 코호트 연구

이 연구는 2005년 구축된 과천시의 4개 초등학교 1학년생 코호트에서 시작되었다. 2008년서울 중구와 경기서남부의 7개교로 규모를 늘린 이래 지속적인 추적조사가 진행되고 있다. 2012년부터 2014년까지 초등학교 고학년과 중고등학생으로 구성된 고도비만 코호트가 별도로 구축되었다.

이 코호트 안에서는 성성숙도 검사와 3일간 24시간 리콜방식을 적용한 식이섭취조사 등이 시행된다. 설문조사에서는 식습관, 건강상태, 운동, 가정환경, 체중조절 등이 조사되고 있다. 소아비만 및 대사질환 코호트 연구는 종단적으로 데이터를 수집하여 소아 비만의 위험요인에 대한 근거를 제공한다. 조사 대상 지역이 일부 지역으로 제한되어 전체 소아인구에 대한 대표성에는 한계점을 지닌다고 볼 수 있다.

어린이 환경보건 출생코호트 연구

이 연구는 환경보건법 제 15조에 근거하고 있다. 질병발생률 산정을 위한 대규모 코호트, 발병원인 규명을 위한 상세코호트로 구분되며 총 10만명 규모의 임신부를 모집하는 것을 목표로 한다. 2015년부터 5년간 7만명을 우선적으로 모집 및 조사하고자 하며 상세코호트에서는 20년간 추적조사가 시행된다.

대규모 코호트에서는 임신부 설문조사, 생체시료 채취, 임상검사, 출생아 추적관찰 등이

Volume: 39, Article ID: e2017059

https://doi.org/10.4178/epih.e2017059 [텍스트 입력]

이루어진다. 상세 코호트에서는 임신부 설문조사 및 환경측정, 혈액 및 요 중 체취 환경 오염물질 분석, 임신부 건강검사, 환경성질환과 유전자간 관련성 확인을 위한 유전자검사, 분만결과 조사, 출생아 추적관찰 등이 시행된다.

어린이 환경보건 출생코호트 연구는 전향적 코호트 연구로 영아부터 청소년기까지 유해 환경요소에 대한 노출과 건강에 미치는 영향을 추적조사하여 노출과 질환간의 인과관계를 밝히고 예방책이 수립될 수 있도록 한다.

청소년 매체이용 및 유해환경 실태조사

청소년 매체이용 및 유해환경 실태조사는 청소년들 주변에서 안전에 위협을 주거나 의식 및 행동에 영향을 줄 수 있는 해로운 환경과 요인들을 파악하고 감시하는 기능의 조사이다. 구체적인 주제로 구성된 이 조사는 학교에 재학 중인 학생과 위기 청소년 모두를 대상으로 시행되고 있다. 여기서 위기 청소년은 지정 청소년 보호시설에 등록된 가출 청소년이나 소년원에 재소 중인 청소년 모두를 포함한다.

청소년 종합실태조사

구체적인 주제로 구성된 다른 청소년관련 설문과 비교하여, 청소년 종합실태조사는 청소년 생활에 영향을 끼칠 수 있는 다양한 영역과 넓은 범위의 주제를 다루고 있다. 설문의 대상자 또한 청소년에서 그치는 것이 아니라, 함께 생활하고 있는 부모님 또는 부양자 모두에게서 설문응답을 받고 있다. 이는 부모님의 역할이나 성장환경이 청소년의 성장과 발달 과정에 매우 중요하게 작용하기 때문이다. 즉, 설문의 대상단위가 청소년 개인이 아닌 등록된 거주지의 한 가정이고, 청소년 대상자는 학교에 재학 중인 청소년과 학교 밖 청소년을 모두 포함한다.

다문화청소년패널조사

우리나라에서 다문화 가정이 차지하는 비중이 증가함에 따라 한국 사회에 다문화적 역량과 사회 통합의 필요성이 요구되고 있다. 이에 다문화청소년패널조사는 다문화배경을 지닌 청소년들의 발달과정을 파악하기 위해 수행되었다.

2010년에 예비조사를 시작하여 2012년까지 1단계 연구를 진행했다. 1단계 연구는 다문화 청소년의 발달과정 종단자료 수집을 목표로 했다. 2단계 연구는 다문화청소년의 발달과정을 추적하고 발달 변화의 요인을 파악하는 것을 목적으로 2013년부터 2017년까지 진행됐다. 단계별 연구 목적에 차이가 있지만, 초기 패널 참여자가 계속 이어지는 동일한 연구의 연속사업이라고 할 수 있다.

Volume: 39, Article ID: e2017059

https://doi.org/10.4178/epih.e2017059 [텍스트 입력]

다문화청소년패널조사는 2011년부터 축적된 패널을 대상으로 설문조사와 면접조사를 실시하였다. 면접조사의 경우, 전체 조사참여자 중 지역별로 4~5 가구를 선정하여 청소년과 어머니를 한 쌍으로 면접하였다.

(Table 3)

자료 접근성

Table 3은 이용 가능한 데이터의 목록이다. 원시데이터는 해당 웹사이트를 통해 얻을 수 있다. 모든 데이터가 공개 되어 있는 것은 아니지만, 모든 통계자료는 주기적인 보고서를 통해 대중에 제공되고 있다.

REFERENCES

1. Korea Centers for Disease Control and Prevention. Analysis of risk factors on obesity and combined metabolic disorders and establishment of cohort in childhood. Cheongju: Korea Centers for Disease Control and Prevention; 2014, p. 6 (Korean, author's translation).

2. World Health Organization. Workshop on capacity-building for health promotion; 2003 [cited 2018 Jan 16]. Available from: http://iris.wpro.who.int/bitstream/handle/10665.1/6052/RS_2002_GE_21_PHL_eng.pdf.

3. Ministry of Environment. Korean Children's Environmental Health Study (I). Sejong: Ministry of Environment; 2016, p. 9 (Korean, author's translation).

4. Korea Centers for Disease Control and Prevention. The 12th data book of Korea Youth Risk Behavior Web-based Survey. Cheongju: Korea Centers for Disease Control and Prevention; 2016, p. 1 (Korean, author's translation).

5. Kim Y, Choi S, Chun C, Park S, Khang YH, Oh K. Data resource profile: the Korea Youth Risk Behavior Web-based Survey (KYRBS). Int J Epidemiol 2016;45:1076-1076e.

6. Korea Institute of Child Care and Education. Panel Study on Korean Children 2015. Seoul: Korea Institute of Child Care and Education; 2015, p. 1-5, 40 (Korean, author's translation).

7. National Youth Policy Institute. The user's guide for 1st-7th research data of Korean Children and Youth Panel Survey. Sejong: National Youth Policy Institute; 2017, p. 1 (Korean, author's translation).

8. Shin HJ. The current status of the Korean student health examination. Korean J Pediatr 2013;56:313-322.

9. Ministry of Gender Equality and Family. The white paper of youth in Korea. Sejong: 2016, p. 139-143 (Korean, author's translation).

10. National Youth Policy Institute. Multicultural Adolescents Panel Study & Research for Policy Making IV. Sejong: National Youth Policy Institute; 2016, p. 3-4 (Korean, author's translation).
